# Transradial Lower Limb Arteriography of the Type Ⅲ Aortic Arch: A Reverse Technique

**DOI:** 10.7759/cureus.78771

**Published:** 2025-02-09

**Authors:** Daisuke Yamazaki, Kazunori Matsumoto, Mitsunori Yuzurihara

**Affiliations:** 1 Cardiology, Akita Cerebrospinal and Cardiovascular Center, Akita, JPN; 2 Radiology, Akita Cerebrospinal and Cardiovascular Center, Akita, JPN

**Keywords:** endovascular interventions, judkins right catheter, lower limb angiography, transradial approach, type 3 aortic arch

## Abstract

Percutaneous coronary intervention is generally performed using the radial artery approach, and in recent years, the radial artery approach has also been used more and more for endovascular treatment. Given this trend, the radial artery approach is also preferable for lower limb angiography when considering patient burden. However, in the case of type III aortic arch morphology, it can be difficult to advance the catheter into the descending aorta using the radial artery approach. In such cases, we have a method for guiding the catheter into the lower limb arteries using the radial artery approach. We were able to perform the procedure without complications in all three cases by reversing the Judkins right 4.0 catheter at the aortic valve, guiding the guidewire to the lower limb artery, and then advancing the catheter to perform lower limb angiography.

## Introduction

Carotid artery intervention is generally performed using the femoral artery approach, but the morphology of the aortic arch affects treatment difficulty. There is a method of classifying the aortic arch morphology used in carotid artery intervention, which classifies it into types I-III according to the height of the aortic arch apex and the origin of the brachiocephalic artery. The higher the type, the more difficult it is to guide the catheter into the carotid artery [1].

In recent years, attempts have been made to use the transradial approach to treat peripheral arterial disease, and reductions in patient burden, such as early bed rest after treatment, have been evaluated [2]. And the development of devices in various sizes has broadened the range of lower extremity lesions that can be treated (e.g., 4-Fr sheathless PV ® {large curve} 115 cm-long guiding sheath {Asahi Intec CO., Ltd., Nagoya, Japan} and Trytop Longbow ® balloon {DVx CO., Ltd., Tokyo, Japan}) [3]. However, when using the transradial approach to guide a guiding catheter into a lower limb artery, the degree of difficulty depends on the morphology of the aortic arch, and aortic arch morphology classification is also being discussed in this field.

We often perform lower limb angiography using the left radial artery (RA) approach during coronary angiography (CAG) and percutaneous coronary intervention (PCI), but we guide the catheter into the lower limb arteries by advancing a 0.035-inch guidewire into the descending aorta using a Judkins right (JR) 4.0-shaped catheter. However, in patients with type III aortic arch, it may not be possible to guide the catheter into the descending aorta using a JR 4.0 catheter. When guiding a catheter into a lower limb artery via the RA approach, it is thought that it is easier to approach via the left RA because the distance from the origin of the subclavian artery to the aortic arch is shorter. However, in the type III aortic arch, the angle between the origin of the left subclavian artery and the aortic arch is acute, making it difficult to guide a guidewire into the aortic arch using a JR 4.0 catheter. In such cases, we have a method for guiding the catheter, and we will introduce three typical cases.

## Case presentation

Case 1

**Figure 1:**
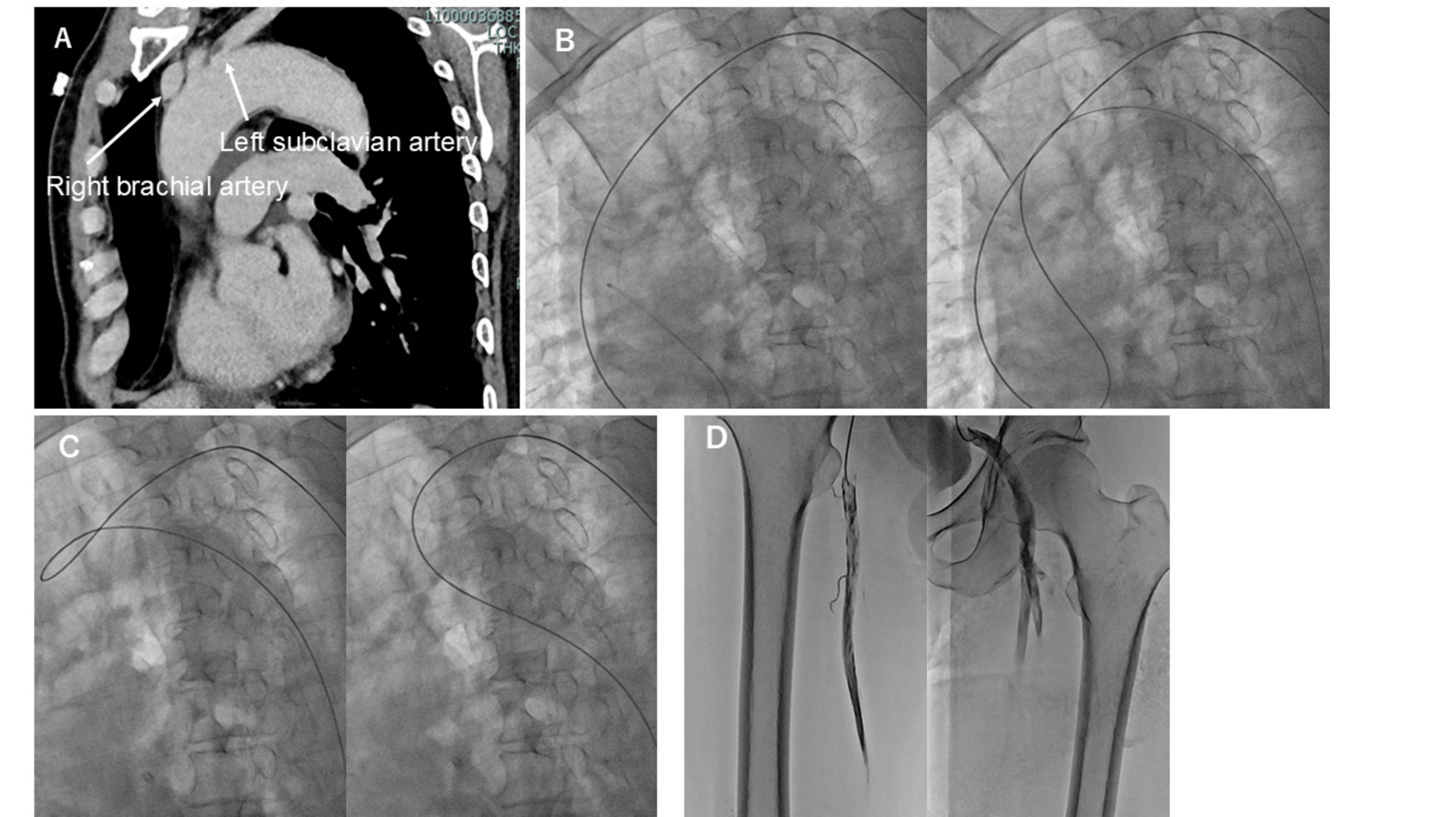
Lower limb angiography in Case 1. (A) The aortic arch is type III on contrast-enhanced computed tomography. (B) The 4-French Judkins right 4.0 catheter was manipulated (left figure) to advance a 0.035-inch guidewire into the descending aorta (right figure). (C) When the catheter was pulled up (left figure), the bend was removed, and the catheter was straightened (right figure). (D) The tip of the catheter was able to be advanced to the superficial femoral artery in the right lower limb (left figure) and to the external iliac artery in the left lower limb (right figure).

A 70-year-old man was admitted to a nearby hospital with congestive heart failure. Echocardiography revealed hypokinesis of the anterior wall, and coronary computed tomography (CT) showed severe stenosis of the right coronary artery (RCA) and left anterior descending artery (LAD). Chest CT at that time confirmed that the aortic arch had a type III morphology (Figure 1A). After the heart failure improved, the patient was referred for PCI. CAG was performed using the left RA approach, and it was found that there was 99% stenosis in the RCA segment (seg) 2 and 90% stenosis in the LAD seg 6, so percutaneous coronary stent implantation was performed on the two lesions. As the patient had good renal function, lower limb angiography was also performed to screen for arteriosclerosis throughout the body. A 4-French (Fr) JR 4.0 catheter (length 120 cm) was used to advance a 0.035-inch guidewire from the left subclavian artery to the descending aorta to perform lower limb angiography, but the aortic arch had a type III morphology, so the guidewire could not be advanced to the descending aorta. Therefore, after inverting the 4-Fr JR 4.0 catheter at the aortic valve, a 0.035-inch guidewire was advanced through the aortic arch into the descending aorta (Figure 1B). After advancing the wire to the abdominal aorta, the catheter was advanced along the wire. When the catheter was pulled up, the deflection of the catheter was removed, and it became possible to directly push the catheter forward (Figure 1C). The catheter tip was advanced to the right superficial femoral artery (SFA) and left external iliac artery (EIA), and lower extremity angiography was performed (Figure 1D). The lower limb angiography was performed without complications, and no stenosis was found in the lower limb arteries. The patient was discharged from the hospital with a good prognosis. The procedure for lower limb angiography in Case 1 is shown in Video 1.

**Video 1:** Lower limb angiography in Case 1. It was not possible to guide the guidewire directly from the origin of the left subclavian artery to the descending aorta using a Judkins right (JR) 4.0 catheter. The JR 4.0 catheter was reversed at the aortic valve, and the guidewire was guided from the aortic arch to the descending aorta. The JR 4.0 catheter was guided to the superficial femoral artery, with the guidewire leading the way.

Case 2

**Figure 2:**
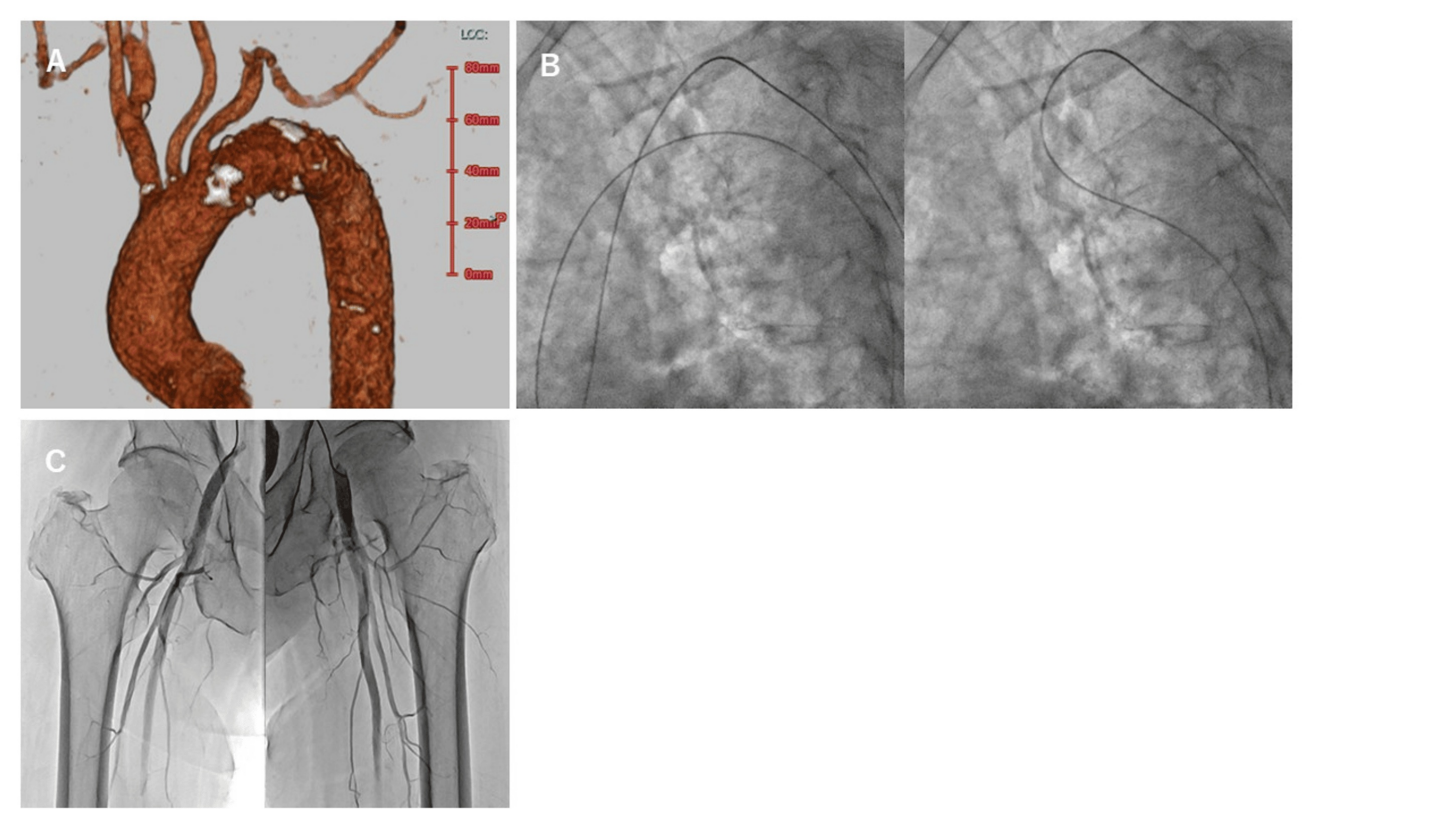
Lower limb angiography in Case 2. (A) The aortic arch is type III on contrast-enhanced computed tomography. (B) A 0.035-inch guidewire was advanced into the descending aorta while being turned around at the aortic valve (left figure). After the 4-French catheter was guided into the descending aorta, it was pulled up, the bend was removed, and it was extended (right figure). (C) The tip of the catheter was advanced to the common femoral artery on both sides.

A 79-year-old man underwent percutaneous coronary stent implantation for acute anterior wall myocardial infarction and was admitted for follow-up CAG one year later. CAG was performed via the left RA approach, and PCI was performed because 90% stenosis was observed in the LAD seg 6. After treatment, lower limb angiography was performed to screen for peripheral arterial disease. A 4-Fr JR 4.0 catheter (length 120 cm) failed to guide a 0.035-inch guidewire directly into the descending aorta. Chest CT performed during a previous coronary CT scan revealed type III aortic arch morphology (Figure 2A). The 0.035-inch guidewire was then reversed at the aortic valve, and it was possible to pass it through the aortic arch. The catheter was guided along the guidewire to the descending aorta. When we pulled the catheter, the bend straightened naturally (Figure 2B). After straightening the deflection, the catheter was pushed forward directly, and the tip was advanced to the bilateral common femoral arteries for contrast imaging (Figure 2C). No stenosis was observed in the lower limb arteries. The procedure was performed without complications, and the patient was discharged after a favorable recovery. The procedure for lower limb angiography in Case 2 is shown in Video 2.

**Video 2:** Lower limb angiography in Case 2. The Judkins right (JR) 4.0 catheter was unable to guide the guidewire directly to the aortic arch. When the guidewire was reversed at the aortic valve, it was able to pass through the aortic arch. When the JR 4.0 catheter was guided to the descending aorta and then pulled up, the catheter straightened out and it was possible to apply direct pushing force.

Case 3

**Figure 3:**
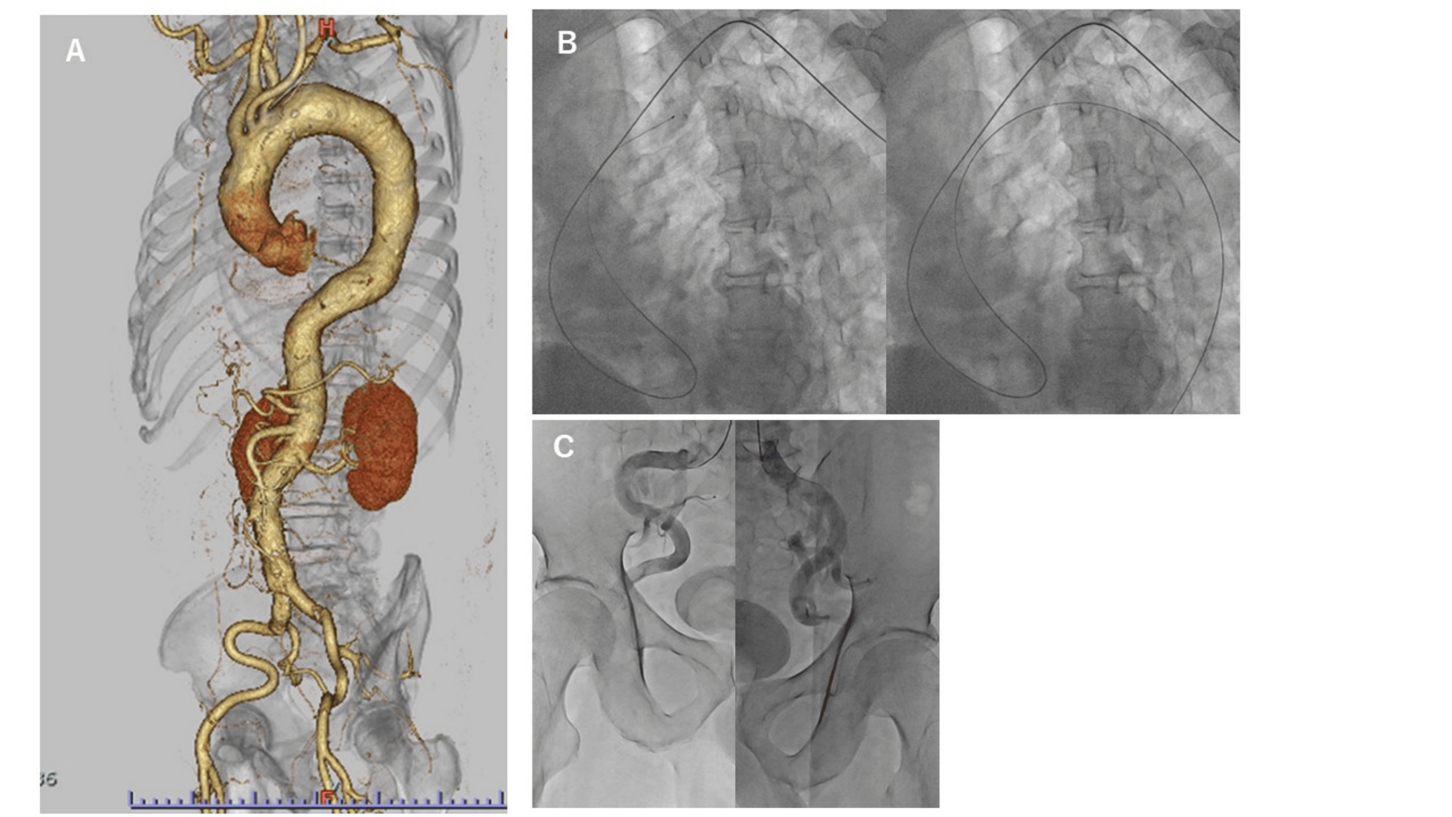
Lower limb angiography in Case 3. (A) The aortic arch is type III on contrast-enhanced computed tomography. The descending aorta is also highly curved. (B) The catheter was advanced in the same way as in case 2, but when it was pulled up and straightened, it bent again when pushed in again. (C) The tip of the catheter could only be advanced to the bilateral common iliac arteries.

A 71-year-old man with a history of cerebral infarction and hypertension presented with an abdominal aortic aneurysm (AAA) on echocardiography. He was followed up with CT, but when the size of the AAA increased to 50 × 52 mm, he underwent CAG for a preoperative examination. CAG was performed via the left RA approach. We then decided to perform lower limb angiography in anticipation of abdominal aortic stent graft implantation. On the previous contrast-enhanced CT scan, the aortic arch was found to have type III morphology with severe curvature of the aorta (Figure 3A). There was no stenosis in the coronary arteries. We then attempted to insert a 0.035-inch guidewire into the descending aorta using a 4-Fr JR 4.0 catheter (120 cm long), but this was difficult because of the shape of the aortic arch. Therefore, when only the 0.035-inch guidewire was reversed at the aortic valve, it was possible to guide the guidewire into the descending aorta (Figure 3B). As in Case 2, the catheter was guided into the descending aorta along the guidewire. The catheter was then pulled up, but the curvature of the aortic arch was so strong that the deflection of the catheter could not be corrected. Therefore, the catheter was guided as far as possible to the periphery while it was still reversed at the aortic valve, and lower limb angiography was performed. Because the catheter was reversed at the aortic valve and the curvature of the aorta was severe, the tip of the catheter could only reach the bilateral common iliac arteries (Figure 3C). The procedure was performed without complications, and the patient was discharged after a favorable recovery. The procedure for lower limb angiography in Case 3 is shown in Video 3.

**Video 3:** Lower limb angiography in Case 3. As in Cases 1 and 2, the catheter was reversed and guided into the lower limb artery, but it was not possible to extend the reversal of the catheter in the ascending aorta. Because the descending aorta was also very curved, the tip of the catheter could only be advanced to the common iliac artery.

## Discussion

Compared with the femoral artery approach, the RA approach has fewer complications, such as bleeding at the puncture site, and allows walking after the procedure [4-6]. Therefore, it has become common to perform PCI using the RA approach. For this reason, it is desirable to perform CAG and lower limb angiography using the RA approach. Coronary angiography is generally performed using JR and Judkins left-shaped catheters. Lower limb angiography is often performed by guiding a guidewire from the left subclavian artery to the descending aorta using a JR catheter. In most cases, this method can be used for lower limb angiography. It is also possible to perform endovascular therapy using the left radial artery approach in most cases [7].

However, when the aortic arch has a type III morphology, the tip of the guidewire may fall into the ascending aorta before it reaches the summit of the aortic arch. In such cases, as in Cases 1 to 3, we inverted the catheter and guidewire at the aortic valve and advanced the guidewire to the distal end of the descending aorta before guiding the catheter. In cases of mild type III aortic arch, the catheter can be advanced from the left subclavian artery to the descending aorta by pulling it up, as in Cases 1 and 2. In such cases, it is easy to guide the catheter into both lower limbs, and with a 120-cm-long catheter, the tip can reach from the external iliac artery to the SFA. If the tip of the catheter can be guided to the external iliac artery, the arteries of the lower limbs can be selectively contrasted, and the internal iliac artery will not be contrasted, so in our experience, contrast can be achieved with about 10 ml of contrast. On the other hand, in cases such as Case 3, in which the aortic arch is highly bent in type III form, even if the catheter is pulled up and straightened, it may fall into the ascending aorta when a pushing force is applied to advance the catheter to the periphery. In such cases, the catheter is advanced to the periphery while being reversed in the ascending aorta. However, with a 120-cm-long catheter, the tip often does not reach the SFA. However, even in cases such as Case 3, where the aorta is strongly bent, and the tip reaches the common iliac artery, it is possible to selectively contrast the arteries of the lower limbs separately on the left and right. The main points to be careful with this method are the risk of plaque embolization due to the force applied to the aortic valve and the risk of acute aortic regurgitation caused by the catheter reversing in the ascending aorta and falling into the left ventricle while the catheter is being advanced through the lower limb arteries. To reduce these risks, we used a thin 4-Fr catheter to reduce the burden on the aortic valve and aortic wall. Of course, there is also the option of trying to pass through the aortic arch using a catheter of a different shape. However, when performing lower limb angiography for the purpose of screening for arteriosclerosis in conjunction with coronary angiography, it is smarter to perform the procedure using a JR 4.0 than to try using various types of catheters.

## Conclusions

In conclusion, this reverse technique allows lower limb angiography to be performed even in cases of type III aortic arch, which were previously difficult to perform using the radial artery approach. In addition, the radial artery approach does not require the patient to rest after the examination, which is expected to reduce the burden on the patient.

## References

[1] Shen S, Jiang X, Dong H (2019). Effect of aortic arch type on technical indicators in patients undergoing carotid artery stenting. J Int Med Res.

[2] Iida O, Takahara M, Fujihara M (2024). Clinical outcomes of transradial vs nontransradial aortoiliac endovascular therapy. JACC Cardiovasc Interv.

[3] Nakamura A, Kanazawa M, Noda K, Endo H, Takahashi T, Nozaki E (2018). Percutaneous transradial artery approach for femoro- popliteal artery intervention in the current era in Japan. Indian Heart J.

[4] Malik AH, Yandrapalli S, Shetty SS (2021). Radial vs. femoral access for percutaneous coronary artery intervention in patients with ST-elevation myocardial infarction. Cardiovasc Revasc Med.

[5] Ahmad F, Usman A, Osama U (2024). Comparison of access site complications in primary percutaneous coronary intervention (PCI) using the radial versus the femoral approach for complex lesions: a prospective study. Cureus.

[6] Anjum I, Khan MA, Aadil M, Faraz A, Farooqui M, Hashmi A (2017). Transradial vs. transfemoral approach in cardiac catheterization: a literature review. Cureus.

[7] Coscas R, de Blic R, Capdevila C, Javerliat I, Goëau-Brissonniere O, Coggia M (2015). Percutaneous radial access for peripheral transluminal angioplasty. J Vasc Surg.

